# Emergency activations for chest pain and ventricular arrhythmias related to regional COVID-19 across the US

**DOI:** 10.1038/s41598-021-03243-6

**Published:** 2021-12-14

**Authors:** Sidney Aung, Eric Vittinghoff, Gregory Nah, Anthony Lin, Sean Joyce, N. Clay Mann, Gregory M. Marcus

**Affiliations:** 1 Division of Cardiology, University of California, 505 Parnassus Ave, M1180B, San Francisco, CA 94143 USA; 2 Department of Epidemiology and Biostatistics, University of California, San Francisco, USA; 3 Department of Medicine, University of California, San Francisco, USA; 4 Department of Pediatrics, University of Utah School of Medicine, Salt Lake City, USA

**Keywords:** Acute coronary syndromes, Arrhythmias, Epidemiology

## Abstract

Evidence that patients may avoid healthcare facilities for fear of COVID-19 infection has heightened the concern that true rates of myocardial infarctions have been under-ascertained and left untreated. We analyzed data from the National Emergency Medical Services Information System (NEMSIS) and incident COVID-19 infections across the United States (US) between January 1, 2020 and April 30, 2020. Grouping events by US Census Division, multivariable adjusted negative binomial regression models were utilized to estimate the relationship between COVID-19 and EMS cardiovascular activations. After multivariable adjustment, increasing COVID-19 rates were associated with less activations for chest pain and non-ST-elevation myocardial infarctions. Simultaneously, increasing COVID-19 rates were associated with more activations for cardiac arrests, ventricular fibrillation, and ventricular tachycardia. Although direct effects of COVID-19 infections may explain these discordant observations, these findings may also arise from patients delaying or avoiding care for myocardial infarction, leading to potentially lethal consequences.

## Introduction

Coronavirus disease 2019 (COVID-19), caused by the severe acute respiratory syndrome coronavirus 2 (SARS-CoV-2), has posed a significant threat to global health. The United States (US) currently leads the world in disease burden with over 33 million documented COVID-19 cases^1^. Although acute COVID-19 has been associated with a systemic inflammatory cytokine response that can directly contribute to coronary artery plaque rupture, activation of procoagulant factors, and hemodynamic changes that may predispose to ischemia, thrombosis, and therefore myocardial infarction^2–4^, several investigators have paradoxically reported marked declines in the incidence of myocardial infarctions during the COVID-19 pandemic^5–10^. Further bolstering these findings, there have been decreasing cardiac catheterization laboratory activations and percutaneous coronary interventions over the same time period^10–12^.

The mechanistic explanations for these dramatic declines in myocardial infarctions remain unknown. One concern has been that those suffering symptoms of myocardial infarctions may be avoiding or postponing visits to a healthcare facility for fear of SARS-CoV-2 exposure, but it is difficult to fully elucidate such a phenomenon at the level of the general population. Consequently, we would anticipate a general decline in presentations to health care for symptoms of myocardial infarctions—namely chest pain.

One of the feared complications of myocardial infarction, particularly when left untreated, are ventricular arrhythmias and cardiac arrests. Studies localized to specific areas experiencing particularly high rates of COVID-19 have demonstrated a rise in out of hospital cardiac arrest, although relationships with myocardial infarctions in those same areas and differences specifically in ventricular fibrillation and ventricular tachycardia have not been described^13–15^. In order to test the hypothesis that increased rates of COVID-19 would be simultaneously associated with a decline in emergency medical services (EMS) activations for chest pain and myocardial infarction with a concomitant rise in activations for cardiac arrests and malignant ventricular arrhythmias, we sought to characterize patterns of each of these phenomena in relationship to SARS-CoV-2 infections throughout the US.

## Results

The relative populations and unadjusted incidence rates at baseline for each of the cardiovascular outcomes of interest prior to the pandemic within each of the 9 US Census Divisions are shown in Table 1.

**Table 1:** Pre-pandemic incidence rates per 10,000 person-years by US Census Division from January 1, 2020 to January 31, 2020.

	Total population (10^7^)	Chest pain	NSTEMI	STEMI	Cardiac arrest	VF	VT
**Divisions**							
New England	0.8	3.5	0.084	0.49	5.2	0.49	0.16
Middle Atlantic	4.1	2.3	0.074	0.55	10.1	0.69	0.29
East North Central	4.7	1.8	0.072	0.45	3.5	0.62	0.17
West North Central	2.1	3.6	0.15	0.51	4.4	0.80	0.22
South Atlantic	6.5	3.9	0.13	0.96	9.1	1.2	0.41
East South Central	1.9	2.4	0.16	0.81	5.1	0.78	0.27
West South Central	4.1	4.7	0.10	0.72	5.9	0.89	0.28
Mountain	2.3	4.3	0.15	0.76	5.8	1.08	0.32
Pacific	5.2	6.3	0.11	0.66	4.2	0.79	0.23

*NSTEMI* non-ST-elevation myocardial infarction, *STEMI* ST-elevation myocardial infarction, *VF* ventricular fibrillation, *VT* ventricular tachycardia.

**Figure 1:**
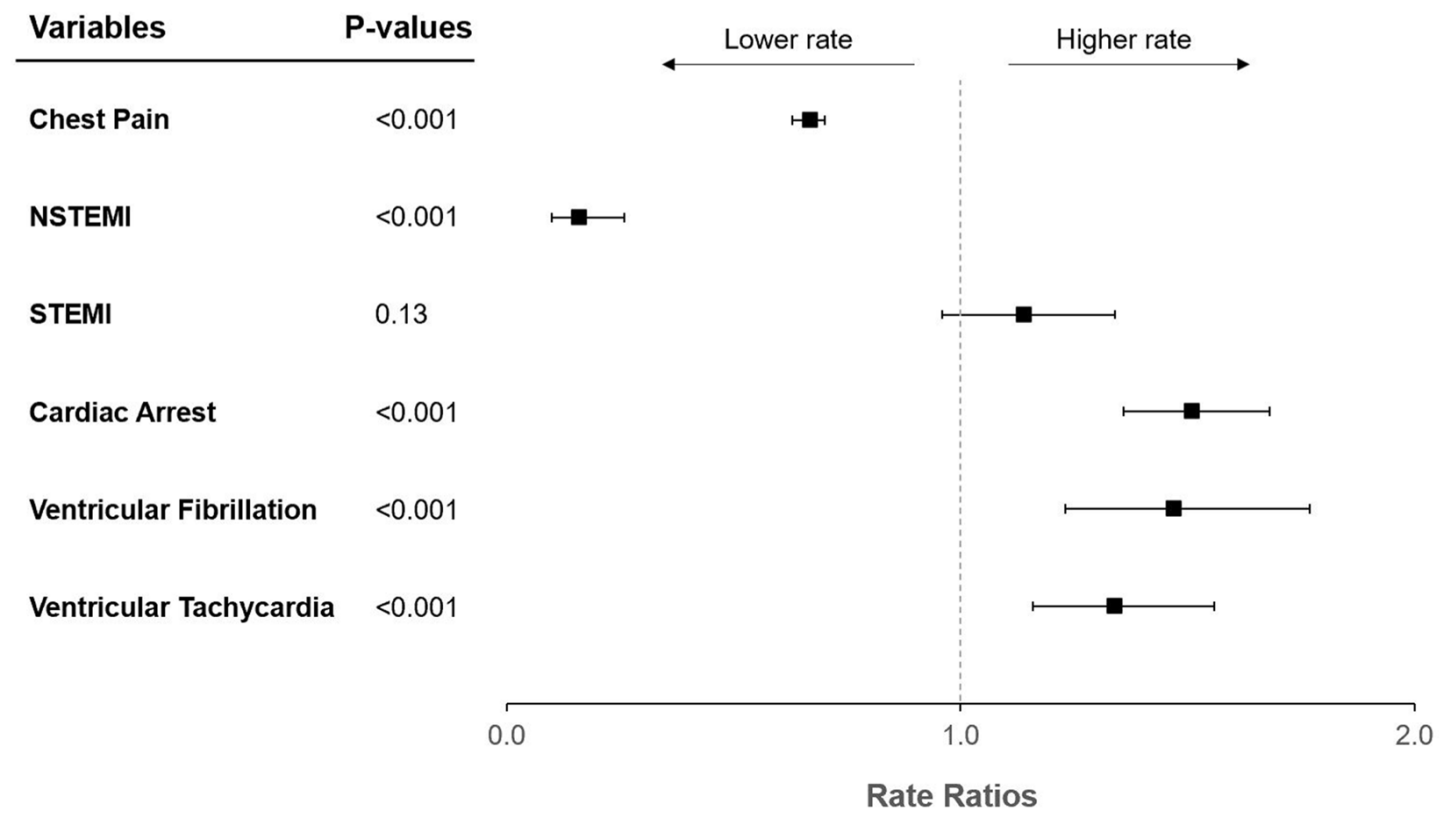
Forest plot of adjusted rate ratios with respect to increasing COVID-19 rates. Rate ratios are interpretable as relative increases in outcome rates per 10,000 person-years for each increase of 10,000 SARS-CoV-2 infections. Y error bars indicate 95% confidence intervals. *COVID-19 * coronavirus disease 2019, *NSTEMI* non-ST-elevation myocardial infarction, *SARS-CoV-2* severe acute respiratory syndrome coronavirus 2, *STEMI* ST-elevation myocardial infarction.

After adjustment for calendar day of week, calendar month, and US Census Division, there was a significant decrease in chest pain events with increasing COVID-19 rates (RR 0.67, 95% CI 0.63–0.70, p < 0.001; Fig. 1). There was also a significant decrease in NSTEMI events with increasing infections (RR 0.16, 95% CI 0.10–0.26, p < 0.001). No statistically significant relationships between the rates of STEMIs and rates of COVID-19 were detected (RR 1.14, 95% CI 0.96–1.34, p = 0.13).

After adjustment for the same covariates and over the same time period, there was a significant increase in cardiac arrest with increasing COVID-19 (RR 1.51, 95% CI 1.36–1.68, p < 0.001; Fig. 1). Similarly, COVID-19 rates were associated with significant increases in ventricular fibrillation (RR 1.47, 95% CI 1.23–1.77, p < 0.001) and ventricular tachycardia (RR 1.34, 95% CI 1.16–1.56, p < 0.001).

**Figure 2:**
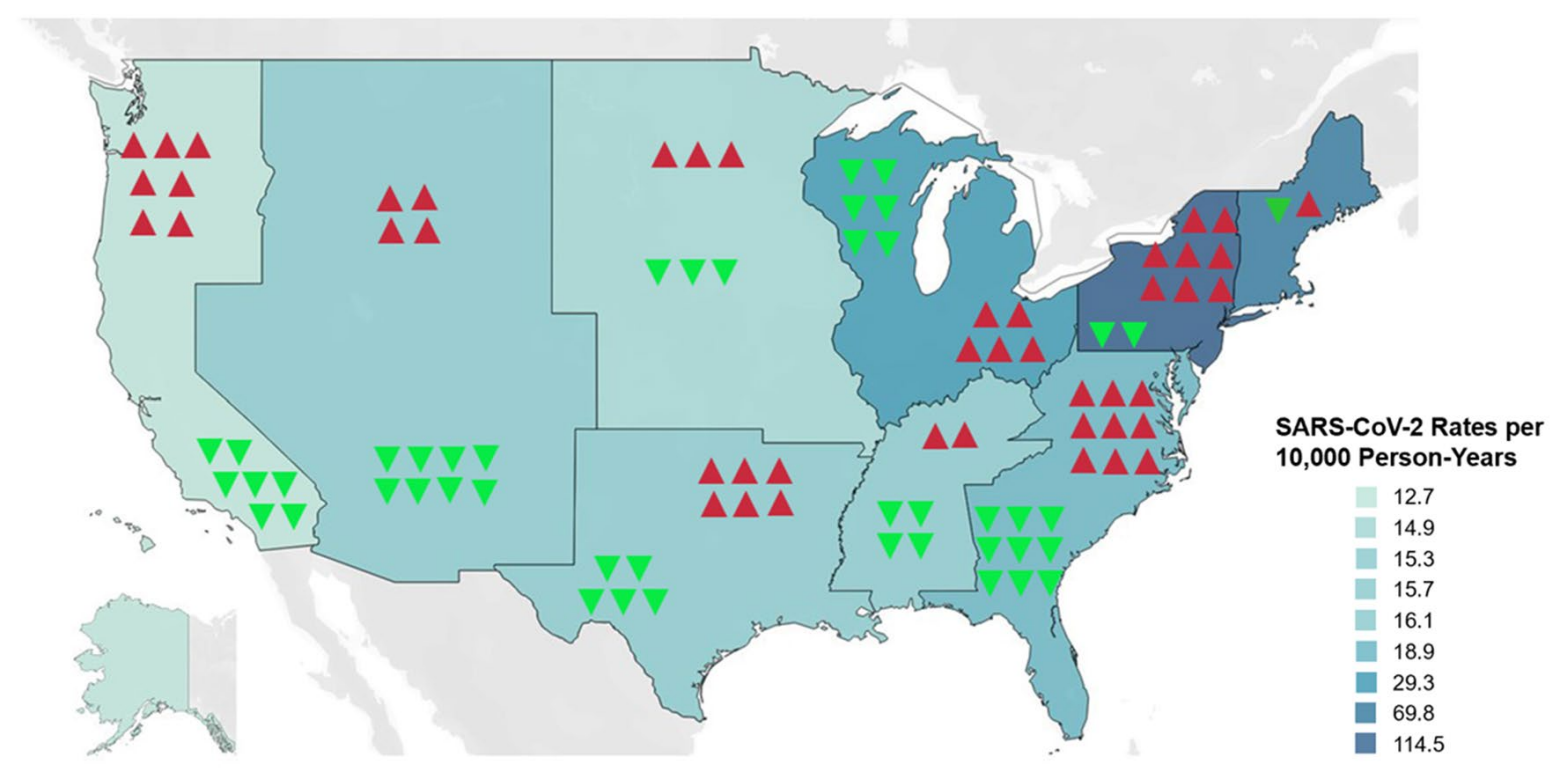
US Census Divisions ranked according to rate differences for NSTEMI and Cardiac Arrest. Blue-teal shading represents gradations scaled to unadjusted SARS-CoV-2 infection rates per 10,000 person-years between January 1, 2020 and April 30, 2020. Downward green arrows represent negative rate differences for NSTEMI. Upward red arrows represent positive rate differences for cardiac arrest. The number of arrows is proportional to the relative magnitude of the rate differences. *NSTEMI* non-ST-elevation myocardial infarction, *SARS-CoV-2* severe acute respiratory syndrome coronavirus 2.

After adjustment for the same covariates, rates of COVID-19 were associated with significant decreases in rate differences for chest pain and NSTEMI (Table 2) along with significant increases in rate differences for cardiac arrest, ventricular fibrillation, and ventricular tachycardia (Table 3) and these findings were seen consistently within each US Census Division (Fig. 2).

**Table 2:** Rate differences by US Census Division for chest pain, NSTEMI, and STEMI.

	Rate difference (chest pain)	95% CI (chest pain)	Rate difference (NSTEMI)	95% CI (NSTEMI)	Rate difference (STEMI)	95% CI (STEMI)
**Divisions**						
New England	− 4705	(− 5337, − 4072)	− 293	(− 406, − 180)	217	(56, 379)
Middle Atlantic	− 10,091	(− 11,458, − 8725)	− 486	(− 672, − 300)	568	(146, 989)
East North Central	− 9153	(− 10,374, − 7931)	− 840	(− 1153, − 527)	733	(189, 1278)
West North Central	− 8066	(− 9144, − 6988)	− 492	(− 678, − 307)	345	(89, 602)
South Atlantic	− 26,764	(− 30,319, − 23,208)	− 1994	(− 2729, − 1260)	2012	(519, 3506)
East South Central	− 4395	(− 4986, − 3804)	− 559	(− 768, − 349)	474	(122, 826)
West South Central	− 18,627	(− 21,106, − 16,148)	− 686	(− 943, − 430)	878	(226, 1530)
Mountain	− 10,825	(− 12,267, − 9383)	− 1365	(− 1868, − 863)	646	(166, 1127)
Pacific	− 39,680	(− 44,939, − 34,420)	− 1190	(− 1631, − 749)	1136	(292, 1980)

Rate differences represent differences in number of outcomes per 10,000 person-years for each increase of 10,000 SARS-CoV-2 infections.

*CI* confidence interval, *NSTEMI* non-ST-elevation myocardial infarction, *SARS-CoV-2* severe acute respiratory syndrome coronavirus 2, *STEMI* ST-elevation myocardial infarction.

**Table 3:** Rate differences by US Census Division for cardiac arrest, ventricular fibrillation, and ventricular tachycardia.

	Rate difference (cardiac arrest)	95% CI (cardiac arrest)	Rate difference (VF)	95% CI (VF)	Rate difference (VT)	95% CI (VT)
**Divisions**						
New England	8099	(7023, 9175)	826	(611, 1041)	207	(101, 313)
Middle Atlantic	32,781	(28,321, 37,240)	2451	(1813, 3089)	732	(358, 1106)
East North Central	20,198	(17,532, 22,864)	2992	(2225, 3759)	678	(334, 1023)
West North Central	10,913	(9470, 12,356)	1829	(1359, 2299)	360	(177, 543)
South Atlantic	69,452	(60,288, 78,616)	7827	(5829, 9825)	2030	(1005, 3055)
East South Central	10,842	(9408, 12,276)	1511	(1121, 1900)	363	(178, 548)
West South Central	24,337	(21,135, 27,539)	3302	(2457, 4147)	841	(415, 1267)
Mountain	15,352	(13,325, 17,378)	2330	(1732, 2928)	640	(315, 966)
Pacific	28,789	(24,996, 32,583)	4681	(3483, 5878)	1167	(576, 1758)

Rate differences represent differences in number of outcomes per 10,000 person-years for each increase of 10,000 SARS-CoV-2 infections.

*CI* confidence interval, *SARS-CoV-2* severe acute respiratory syndrome coronavirus 2, *VF* ventricular fibrillation, *VT* ventricular tachycardia.

**Figure 3:**
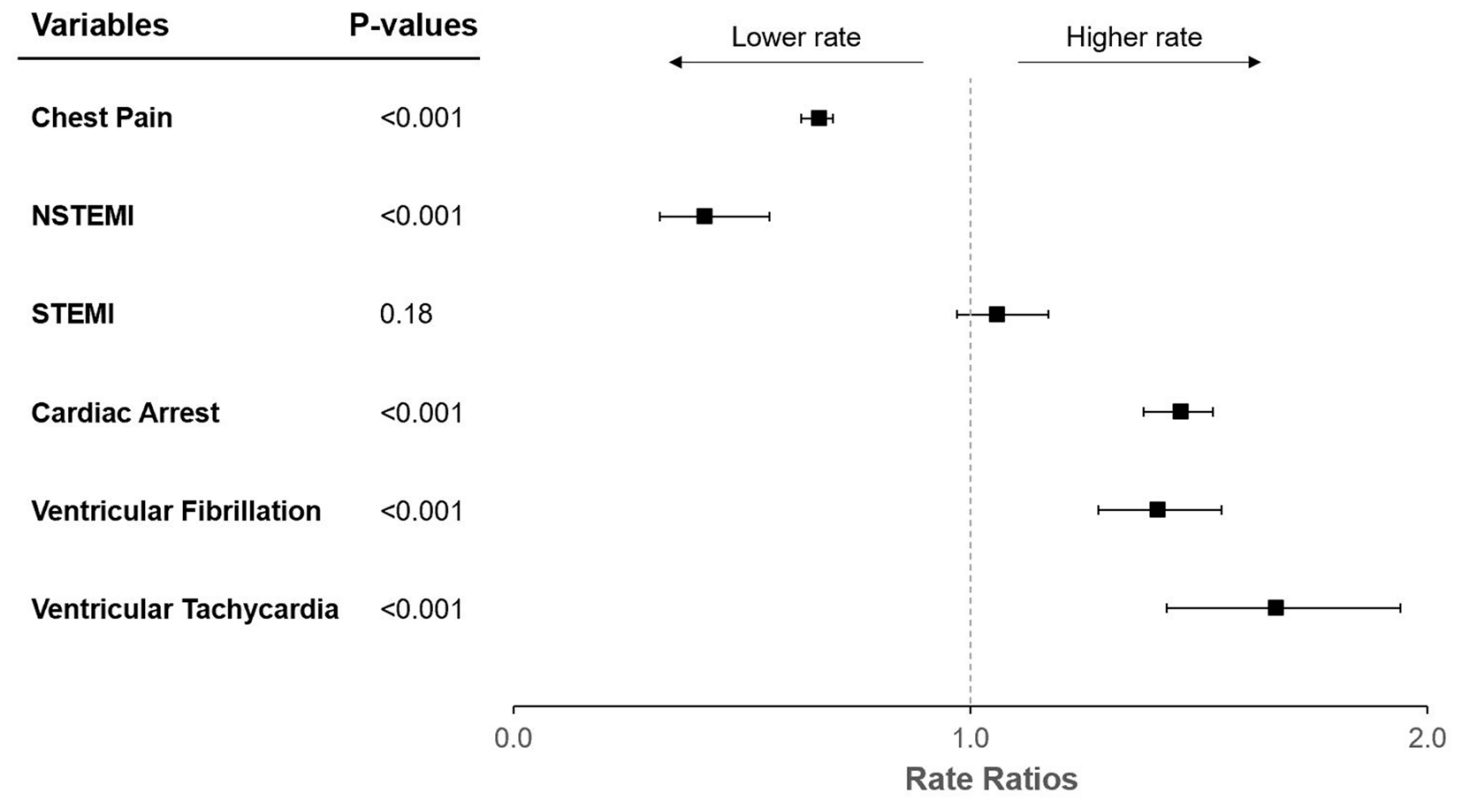
Forest plot of adjusted rate ratios with respect to increasing COVID-19 rates after excluding EMS activations with concurrent COVID-19 signs and symptoms. Rate ratios are interpretable as relative increases in outcome rates per 10,000 person-years for each increase of 10,000 SARS-CoV-2 infections. *COVID-19* coronavirus disease 2019, *NSTEMI* non-ST-elevation myocardial infarction, *SARS-CoV-2* severe acute respiratory syndrome coronavirus 2, *STEMI* ST-elevation myocardial infarction. Y error bars indicate 95% confidence intervals.

In sensitivity analyses excluding all cases with EMS impressions for fever, sepsis, pneumonia, respiratory distress, or respiratory failure, none of the results were meaningfully different (Fig. 3, Supplementary Tables 1–3).

## Discussion

In this national sample, as COVID-19 rates rose, chest pain and NSTEMI cases fell. No statistically significant relationship between COVID-19 rates and STEMI was observed. However, simultaneous with these phenomena, cardiac arrests and cases of both ventricular fibrillation and ventricular tachycardia each significantly increased concomitant with an increasing incidence of COVID-19.

Our findings largely fit with previous reports describing a decreasing incidence of myocardial infarctions during the pandemic^5–10^. Unlike some studies limited to smaller regions of the US reporting substantial declines in STEMI, we did not observe a similar statistically significant relationship in this national evaluation. Interestingly, previous investigators have described a larger magnitude in reductions in NSTEMI than STEMI associated with the pandemic^6–10^, consistent with our results.

The reasons for the differential relationships between STEMI and NSTEMI as well as the general trends favoring reductions in these outcomes remain unclear. Indeed, COVID-19 is associated with coronary artery plaque rupture^2–4^ and has been implicated as a primary cause of myocardial infarctions in some cases^16,17^. It is possible there is some generalized effect related to the pandemic, perhaps related to shelter-in-place orders, that has resulted in an overall reduced propensity to myocardial infarction.

An alternative explanation is that there is no true reduction in myocardial infarctions, but that instead these studies as well as our current analysis all suffer from under-ascertainment because we are all relying on myocardial infarction patients seeking medical attention and being “counted”. The current study focused solely on EMS activations for outcomes such as myocardial infarctions, and thus does not reflect the “true” rate of these events occurring in the population. COVID-19-related fear, specifically the prospect of becoming infected (and then perhaps infecting loved ones) may reduce visits to healthcare facilities^18–20^ and delay care for myocardial infarctions^21^. Given the greater severity of STEMI, usually with more pronounced symptoms that may be more difficult to ignore or minimize, it appears plausible that a general reluctance to seek medical attention due to fear of COVID-19 may disproportionately affect those experiencing NSTEMI, fitting with the discrepant observations between SARS-CoV-2 related to STEMI and NSTEMI as others, and now we, have observed.

If in fact the true incidence of NSTEMI was not decreasing, but instead individuals suffering from NSTEMI were simply not presenting to clinical attention, we would anticipate less ambulance calls for chest pain as demonstrated in the current study. Indeed, there is evidence that EMS activations in general have decreased across the United States during the early pandemic^22^, indicating that this is a broad phenomenon. Furthermore, one might expect to observe increases in the adverse events that would result from untreated myocardial infarctions. If indeed the suppression of healthcare utilization due to COVID-19-related concern was operative, presumably only the most severe and consequential events would result in those same patients ultimately seeking medical attention.

And indeed, during this same time period, despite the apparent decrease in chest pain and NSTEMI, we observed significant increases in cardiac arrests, ventricular fibrillation, and ventricular tachycardia with rising rates of SARS-CoV-2 infections. It is possible that direct effects related to the virus also played a role here. Those hospitalized with severe COVID-19 have been shown to experience a substantially increased rate of lethal ventricular arrhythmias^23–25^. There are various potential mechanisms linking COVID-19 to arrhythmogenesis including hypoxia, myocarditis, abnormal host immune response, myocardial ischemia, myocardial strain, electrolyte derangements, intravascular volume imbalances, and drug sides effects^26^. Cardiac MRI has also revealed myocardial delayed enhancement indicative of scar in large proportions of COVID-19 patients^27,28^, potentially providing a cardiac substrate particularly prone to ventricular arrhythmias and cardiac arrest. However, one can argue that the scale of the increases in the number of the outcomes observed in the current national study is too large for these changes to be primarily attributable to the virus itself. Furthermore, there is evidence that atrial arrhythmias, not a focus of the current study, are the most common cardiac arrhythmia observed in COVID-19 patients^29,30^. It is difficult, if not impossible, to glean effects directly related to viral infection from those that may have occurred due to untreated myocardial infarctions, and a combination of both may also be present. Of note, in our sensitivity analyses excluding primary EMS impressions of respiratory or infectious phenomena occurring in the EMS activations, none of our findings were meaningfully changed.

Several limitations of the current study should be acknowledged. Given the ecologic study design, there is the risk for the “ecological fallacy”, wherein we must acknowledge that aggregated population data may not accurately reflect purported mechanisms or intentions at the individual level. However, inferring the nature of the effects of interest in the current study required an assessment of particularly large numbers of people over heterogenous and broad geographic regions, making an ecologic study design appropriate for the current circumstances. Because geographic identifiers below US Census Division were not available for public use in the NEMSIS dataset, we were unable to capture differences at the state, county, or city level that may yet be important. However, absent meaningful interactions related to these more granular locations and the outcomes studied, we do not believe this limitation should have created spurious false positive results. We relied on the judgment and experience of EMS personnel to correctly report chest pain, identify myocardial infarctions, and interpret electrocardiograms and physical examination results (such as the lack of a pulse) to determine the diagnoses of interest. Given that patients with myocardial infarctions may present with symptoms other than chest pain, the observed decline in chest pain associated with increasing COVID-19 rates may have been due to EMS personnel reporting disproportionately more concurrent symptoms such as shortness of breath due to the impact of the ongoing COVID-19 pandemic. While evidence suggests that EMS personnel are generally accurate in identifying STEMIs^31–34^, the accurate diagnoses of NSTEMI is more nuanced and therefore may be more prone to error. While distinguishing ventricular tachycardia from supraventricular tachycardia can be difficult, diagnoses of ventricular fibrillation and cardiac arrest clearly fall within the EMS personnel’s area of expertise. Regardless, it would appear unlikely that EMS personnel nationally—and within each separate US Census Division alone—would both be less likely to make a diagnosis of NSTEMI and yet more likely report diagnoses of cardiac arrest, ventricular fibrillation, and ventricular tachycardia as rates of SARS-CoV-2 changed in their region. And finally, chest pain, a subjective symptom that clearly decreased with SARS-CoV-2 rates, is essentially defined by the complaint offered by the patient without substantial skill required of the evaluating EMS personnel.

## Conclusions

In conclusion, regional COVID-19 rates were associated with a decline in EMS activations for chest pain and NSTEMI along with a simultaneous rise in activations for cardiac arrests and malignant ventricular arrhythmias. Although effects of SARS-CoV-2 infections may explain these discordant observations, these findings may also arise from patients delaying or avoiding care for myocardial infarction, leading to potentially lethal consequences. Public health interventions aimed at reinforcing the need to seek appropriate care for suspected myocardial infarctions, especially in the highest risk patients, should be pursued. While the prevention of contracting and transmitting COVID-19 is important, plans and resources must also be in place to reduce the impact on the timely diagnosis and treatment of other serious conditions.

## Methods

### Study design

We conducted an ecological study to investigate the association between rates of COVID-19 and EMS activations for chest pain, myocardial infarction, cardiac arrest, and malignant ventricular arrhythmia between January 1, 2020 and April 30, 2020 across the US. The study period was chosen given that the first SARS-CoV-2 infections were recorded in January and continued until the end of the most recent data for EMS activation was available. The period beginning in March corresponds to the timing of the first major surge of SARS-CoV-2 infections in the US^1^.

We abstracted the daily number of events for chest pain, non-ST-elevation myocardial infarction (NSTEMI), ST-elevation myocardial infarctions (STEMI), cardiac arrests, ventricular fibrillation, and ventricular tachycardia from the National Emergency Medical Services Information System (NEMSIS) database. NEMSIS is a national EMS registry that includes standardized patient care records submitted by over 10,000 EMS agencies across 47 states and territories in near real-time^35^. Most states require all EMS-related activations to be documented in NEMSIS. Additional information regarding NEMSIS and its submitting agencies can be obtained from the NEMSIS Technical Assistance Center and from previous studies that have utilized the NEMSIS database^36–38^. Diagnoses were recorded during an event by EMS personnel based on symptoms, physical examination (such as whether a pulse was detected), and electrocardiographic tracings. Geographic location for each event was aggregated into each of the nine US Census Divisions (https://www.ncdc.noaa.gov/monitoring-references/maps/us-census-divisions.php). Requests to access the data can be made through the NEMSIS registry after submitting a proposal: https://nemsis.org/using-ems-data/request-research-data.

We used epidemiological data from the Johns Hopkins University Center of Systems Science and Engineering COVID-19 dashboard to retrieve information regarding SARS-CoV-2 infections by location across all US states^1,39^. State-level counts of SARS-CoV-2 infections were aggregated into their respective US Census Divisions to match the NEMSIS outcomes data. We used 2019 US Census data to obtain population denominators and calculate incidence rates.

Certification to use de-identified NEMSIS data was obtained from the University of California, San Francisco Institutional Review Board.

### Statistical analysis

Negative binomial regression models were used to estimate the relationship between the daily rate of SARS-CoV-2 infections and the time-matched daily rate of EMS activations for chest pain, myocardial infarctions, cardiac arrest, and ventricular arrhythmias within the same US Census Divisions. We adjusted for calendar day of week, calendar month, and US Census Division in our models to derive the following adjusted measures of association. We derived estimates for incidence rate ratios (RR) for our outcomes, which are interpretable as relative increases in outcome rates per 10,000 person-years for each increase of 10,000 SARS-CoV-2 infections. We also derived estimates for rate differences, which are interpretable as differences in number of outcomes per 10,000 person-years for each increase of 10,000 SARS-CoV-2 infections. We performed sensitivity analyses after excluding all cases with signs or symptoms suggestive of active viral and/ or respiratory infection, including fever, sepsis, pneumonia, respiratory distress, and respiratory failure. Statistical analyses were performed using Stata, version 16 (College Station, TX, USA). Two-tailed p values < 0.05 were considered statistically significant.

## Supplementary Information

Supplementary Information.

## Data Availability

Requests to access the data can be made through the NEMSIS registry after submitting a proposal: https://nemsis.org/using-ems-data/request-research-data.

## References

[1] Johns Hopkins University. Coronavirus COVID-19 global cases. https://coronavirus.jhu.edu/map.html. Published 2020. Accessed June 24, 2021.

[2] Zhou F (2020). Clinical course and risk factors for mortality of adult inpatients with COVID-19 in Wuhan, China: A retrospective cohort study. Lancet.

[3] Driggin E (2020). Cardiovascular considerations for patients, health care workers, and health systems during the COVID-19 pandemic. J. Am. Coll. Cardiol..

[4] Kermani-Alghoraishi M (2021). A review of coronary artery thrombosis: A new challenging finding in COVID-19 patients and ST-elevation myocardial infarction. Curr. Probl. Cardiol..

[5] Solomon MD (2020). The Covid-19 pandemic and the incidence of acute myocardial infarction. N. Engl. J. Med..

[6] De Filippo O (2020). Reduced rate of hospital admissions for ACS during Covid-19 outbreak in Northern Italy. N. Engl. J. Med..

[7] De Rosa S (2020). Reduction of hospitalizations for myocardial infarction in Italy in the COVID-19 era. Eur. Heart J..

[8] Metzler B, Siostrzonek P, Binder RK, Bauer A, Reinstadler SJ (2020). Decline of acute coronary syndrome admissions in Austria since the outbreak of COVID-19: The pandemic response causes cardiac collateral damage. Eur. Heart J..

[9] Mesnier J (2020). Hospital admissions for acute myocardial infarction before and after lockdown according to regional prevalence of COVID-19 and patient profile in France: A registry study. Lancet Public Health.

[10] Mafham MM (2020). COVID-19 pandemic and admission rates for and management of acute coronary syndromes in England. Lancet.

[11] Garcia S (2020). Reduction in ST-segment elevation cardiac catheterization laboratory activations in the United States during COVID-19 pandemic. J. Am. Coll. Cardiol..

[12] Garcia S (2020). Impact of COVID-19 pandemic on STEMI care: An expanded analysis from the United States [published online ahead of print, 2020 Aug 7]. Catheter. Cardiovasc. Interv..

[13] Lai PH (2020). Characteristics associated with out-of-hospital cardiac arrests and resuscitations during the novel coronavirus disease 2019 pandemic in New York City [published online ahead of print, 2020 Jun 19]. JAMA Cardiol..

[14] Marijon E (2020). Out-of-hospital cardiac arrest during the COVID-19 pandemic in Paris, France: A population-based, observational study. Lancet Public Health.

[15] Baldi E (2020). Out-of-hospital cardiac arrest during the Covid-19 outbreak in Italy. N. Engl. J. Med..

[16] Rey JR (2020). COVID-19 and simultaneous thrombosis of two coronary arteries. Rev. Esp. Cardiol. (Engl. Ed.).

[17] Dominguez-Erquicia P, Dobarro D, Raposeiras-Roubín S, Bastos-Fernandez G, Iñiguez-Romo A (2020). Multivessel coronary thrombosis in a patient with COVID-19 pneumonia. Eur. Heart J..

[18] Gorini F (2020). "Acute myocardial infarction in the time of COVID-19": A review of biological, environmental, and psychosocial contributors. Int. J. Environ. Res. Public Health.

[19] Torales J, O'Higgins M, Castaldelli-Maia JM, Ventriglio A (2020). The outbreak of COVID-19 coronavirus and its impact on global mental health. Int. J. Soc. Psychiatry.

[20] Ornell F, Schuch JB, Sordi AO, Kessler FHP (2020). "Pandemic fear" and COVID-19: Mental health burden and strategies. Braz. J. Psychiatry.

[21] Hammad TA (2021). Impact of COVID-19 pandemic on ST-elevation myocardial infarction in a non-COVID-19 epicenter. Catheter. Cardiovasc. Interv..

[22] Lerner EB, Newgard CD, Mann NC (2020). Effect of the coronavirus disease 2019 (COVID-19) pandemic on the US emergency medical services system: A preliminary report. Acad. Emerg. Med..

[23] Guo T (2020). Cardiovascular implications of fatal outcomes of patients with coronavirus disease 2019 (COVID-19). JAMA Cardiol..

[24] Si D (2020). Death, discharge and arrhythmias among patients with COVID-19 and cardiac injury. CMAJ.

[25] Bhatla A (2020). COVID-19 and cardiac arrhythmias. Heart Rhythm.

[26] Dherange P, Lang J, Qian P (2020). Arrhythmias and COVID-19: A review. JACC Clin. Electrophysiol..

[27] Puntmann VO (2020). Outcomes of cardiovascular magnetic resonance imaging in patients recently recovered from coronavirus disease 2019 (COVID-19). JAMA Cardiol..

[28] Huang L (2020). Cardiac involvement in patients recovered from COVID-2019 identified using magnetic resonance imaging. JACC Cardiovasc. Imaging.

[29] Coromilas EJ, Kochav S, Goldenthal I (2021). Worldwide survey of COVID-19-associated arrhythmias. Circ. Arrhythm. Electrophysiol..

[30] Gopinathannair R, Merchant FM, Lakkireddy DR (2020). COVID-19 and cardiac arrhythmias: A global perspective on arrhythmia characteristics and management strategies. J. Interv. Card. Electrophysiol..

[31] Whitbread M, Leah V, Bell T, Coats TJ (2002). Recognition of ST elevation by paramedics. Emerg. Med. J..

[32] Ducas RA (2012). To transmit or not to transmit: How good are emergency medical personnel in detecting STEMI in patients with chest pain?. Can. J. Cardiol..

[33] Davis DP (2007). The positive predictive value of paramedic versus emergency physician interpretation of the prehospital 12-lead electrocardiogram. Prehosp. Emerg. Care.

[34] Trivedi K, Schuur JD, Cone DC (2009). Can paramedics read ST-segment elevation myocardial infarction on prehospital 12-lead electrocardiograms?. Prehosp. Emerg. Care..

[35] NEMSIS. State map v3. https://nemsis.org/state-data-managers/state-map-v3/. Published 2020. Accessed October 12, 2020.

[36] Hsia RY (2018). A US national study of the association between income and ambulance response time in cardiac arrest. JAMA Netw. Open.

[37] Byrne JP (2019). Association between emergency medical service response time and motor vehicle crash mortality in the United States. JAMA Surg..

[38] Kleinman ME (2015). Part 5: Adult basic life support and cardiopulmonary resuscitation quality: 2015 American Heart Association guidelines update for cardiopulmonary resuscitation and emergency cardiovascular care. Circulation.

[39] Dong E, Du H, Gardner L (2020). An interactive web-based dashboard to track COVID-19 in real time [published correction appears in Lancet Infect Dis. 2020 Sep;20(9):e215]. Lancet Infect. Dis..

