# Supplementary material for: Emergency activations for chest pain and ventricular arrhythmias related to regional COVID-19 across the US
Source: Sci Rep. 2021 Dec 14;11:23959. doi: 10.1038/s41598-021-03243-6 (PMC8671431; doi:10.1038/s41598-021-03243-6)
Supplement: Supplementary file 1 — Supplementary Information. [file 41598_2021_3243_MOESM1_ESM.docx]

**Emergency Activations for Chest Pain and Ventricular Arrhythmias Related to Regional COVID-19 Across the US**

Sidney Aung, BA,^1^ Eric Vittinghoff, PhD,^2^ Gregory Nah, MA,^1^ Anthony Lin, MD,^3^ Sean Joyce, BS,^1^ N. Clay Mann, PhD, MS,^4^ *Gregory M Marcus, MD, MAS^1^

^1^Division of Cardiology, University of California, San Francisco. ^2^Department of Epidemiology and Biostatistics, University of California, San Francisco. ^3^Department of Medicine, University of California, San Francisco. ^4^Department of Pediatrics, University of Utah School of Medicine.

**Supplementary Table 1. Relative frequencies of EMS activations with concurrent COVID-19 signs and symptoms.**

|  | **NSTEMI**  N = 2,781 | **STEMI**  N = 19,188 | **Cardiac Arrest**  N = 148,915 | **VF**  N = 14,827 | **VT**  N = 6,085 |
| --- | --- | --- | --- | --- | --- |
|  |  |  |  |  |  |
| **Fever**, n (%) | 4 (0.14) | 26 (0.14) | 49 (0.03) | 2 (0.01) | 10 (0.16) |
| **Sepsis**, n (%) | 12 (0.43) | 53 (0.28) | 113 (0.08) | 3 (0.02) | 22 (0.36) |
| **Pneumonia**, n (%) | 10 (0.36) | 18 (0.09) | 49 (0.03) | 4 (0.03) | 6 (0.10) |
| **Respiratory Distress**, n (%) | 72 (2.59) | 398 (2.07) | 1120 (0.75) | 83 (0.56) | 130 (2.14) |
| **Respiratory Failure**, n (%) | 5 (0.18) | 39 (0.20) | 1379 (0.93) | 108 (0.73) | 48 (0.79) |
| COVID-19 indicates coronavirus disease 2019; N, total number of EMS visits for each respective outcome; n (%), absolute number (percentage) of each concurrent COVID-19 sign and symptom; NSTEMI, non-ST-Elevation myocardial infarction; STEMI, ST-Elevation myocardial infarction; VF, ventricular fibrillation; and VT, ventricular tachycardia. | | | | | |

**Supplementary Table 2. Rate differences by US Census Division for chest pain, NSTEMI, and STEMI after excluding EMS activations with concurrent COVID-19 signs and symptoms.**

|  | **Rate**  **Difference**  **(Chest Pain)** | **95% CI**  **(Chest Pain)** | **Rate**  **Difference**  **(NSTEMI)** | **95% CI**  **(NSTEMI)** | **Rate**  **Difference**  **(STEMI)** | **95% CI**  **(STEMI)** |
| --- | --- | --- | --- | --- | --- | --- |
| **Divisions** |  |  |  |  |  |  |
| New England | -4705 | (-5337, -4072) | -348 | (-468, -229) | 106 | (-50, 261) |
| Middle Atlantic | -10091 | (-11458, -8725) | -568 | (-762, -374) | 269 | (-126, 664) |
| East North Central | -9153 | (-10374, -7931) | -1001 | (-1331, -671) | 356 | (-166, 878) |
| West North Central | -8066 | (-9144, -6988) | -579 | (-772, -385) | 168 | (-79, 416) |
| South Atlantic | -26764 | (-30319, -23208) | -2346 | (-3108, -1585) | 969 | (-453, 2390) |
| East South Central | -4395 | (-4986, -3804) | -669 | (-891, -446) | 232 | (-108, 572) |
| West South Central | -18627 | (-21106, -16148) | -800 | (-1065, -535) | 419 | (-196, 1034) |
| Mountain | -10825 | (-12267, -9383) | -1641 | (-2172, -1109) | 315 | (-147, 776) |
| Pacific | -39680 | (-44939, -34420) | -1423 | (-1888, -959) | 536 | (-251, 1322) |
| Rate differences represent differences in number of outcomes per 10,000 person-years for each increase of 10,000 SARS-CoV-2 infections.  CI indicates confidence interval; COVID-19, coronavirus disease 2019; NSTEMI, non-ST-elevation myocardial infarction; SARS-CoV-2, severe acute respiratory syndrome coronavirus 2; and STEMI, ST-elevation myocardial infarction. | | | | | | |

**Supplementary Table 3. Rate differences by US Census Division for cardiac arrest, ventricular fibrillation, and ventricular tachycardia after excluding EMS activations with concurrent COVID-19 signs and symptoms.**

|  | **Rate**  **Difference**  **(Cardiac Arrest)** | **95% CI**  **(Cardiac Arrest)** | **Rate**  **Difference**  **(VF)** | **95% CI**  **(VF)** | **Rate**  **Difference**  **(VT)** | **95% CI**  **(VT)** |
| --- | --- | --- | --- | --- | --- | --- |
| **Divisions** |  |  |  |  |  |  |
| New England | 6574 | (5637, 7511) | 720 | (513, 926) | 305 | (208, 402) |
| Middle Atlantic | 27110 | (23193, 31027) | 2058 | (1468, 2649) | 1010 | (691, 1329) |
| East North Central | 16997 | (14591, 19404) | 2601 | (1863, 3339) | 929 | (643, 1215) |
| West North Central | 9060 | (7775, 10344) | 1509 | (1079, 1938) | 512 | (353, 672) |
| South Atlantic | 56986 | (48920, 65052) | 6726 | (4823, 8629) | 2707 | (1887, 3527) |
| East South Central | 9049 | (7766, 10332) | 1296 | (927, 1666) | 523 | (360, 686) |
| West South Central | 20181 | (17332, 23030) | 2829 | (2027, 3631) | 1145 | (794, 1495) |
| Mountain | 12684 | (10888, 14479) | 1990 | (1424, 2555) | 891 | (617, 1166) |
| Pacific | 23363 | (20059, 26666) | 3933 | (2818, 5048) | 1537 | (1067, 2006) |
| Rate differences represent differences in number of outcomes per 10,000 person-years for each increase of 10,000 SARS-CoV-2 infections.  CI indicates confidence interval; COVID-19, coronavirus disease 2019; SARS-CoV-2, severe acute respiratory syndrome coronavirus 2; VF, ventricular fibrillation; and VT, ventricular tachycardia. | | | | | | |
